# Cannabis-Based Medicine for Neuropathic Pain and Spasticity—A Multicenter, Randomized, Double-Blinded, Placebo-Controlled Trial

**DOI:** 10.3390/ph16081079

**Published:** 2023-07-28

**Authors:** Julie Schjødtz Hansen, Stefan Gustavsen, Homayoun Roshanisefat, Matthias Kant, Fin Biering-Sørensen, Claus Andersen, Anna Olsson, Helene Højsgaard Chow, Nasrin Asgari, Julie Richter Hansen, Helle Hvilsted Nielsen, Rikke Middelhede Hansen, Thor Petersen, Annette Bang Oturai, Finn Sellebjerg, Eva Aggerholm Sædder, Helge Kasch, Peter Vestergaard Rasmussen, Nanna Brix Finnerup, Kristina Bacher Svendsen

**Affiliations:** 1 Department of Neurology, Aarhus University Hospital (AUH), 8200 Aarhus, Denmark; 2 Department of Clinical Medicine, Aarhus University, 8200 Aarhus, Denmark; 3 Danish Multiple Sclerosis Center (DMSC), Department of Neurology, Copenhagen University Hospital, Rigshospitalet, 2600 Glostrup, Denmark; 4 Department of Neurology, Naestved, Slagelse & Ringsted Hospitals, Region Zealand, 4200 Slagelse, Denmark; 5 Department of Neurology, Hospital of Southern Jutland, 6400 Soenderborg, Denmark; 6 Department of Neurology, Hospital South-West Jutland Esbjerg, 6700 Esbjerg, Denmark; 7 Department of Brain and Spinal Cord Injuries, Copenhagen University Hospital, Rigshospitalet, 2600 Glostrup, Denmark; 8 Institute of Regional Health Research and Molecular Medicine, University of Southern Denmark, 5000 Odense, Denmark; 9 Department of Neurology, Herlev Hospital, 2730 Herlev, Denmark; 10 Department of Neurology, Odense University Hospital, 5000 Odense, Denmark; 11 Spinal Cord Injury Centre of Western Denmark (SCIWDK), Viborg Regional Hospital, 8800 Viborg, Denmark; 12 Department of Neurology, Hospital of Southern Jutland and Research Unit in Neurology, Department of Regional Health Research, University of Southern Denmark, 5000 Odense, Denmark; 13 Department of Clinical Pharmacology, Aarhus University Hospital, 8000 Aarhus, Denmark; 14 Danish Pain Research Centre, Aarhus University, 8200 Aarhus, Denmark

**Keywords:** cannabis-based medicine (CBM), delta-9-tetrahydrocannabinol (THC), cannabidiol (CBD), neuropathic pain (NP), spasticity

## Abstract

Patients with multiple sclerosis (MS) and spinal cord injury (SCI) commonly sustain central neuropathic pain (NP) and spasticity. Despite a lack of consistent evidence, cannabis-based medicine (CBM) has been suggested as a supplement treatment. We aimed to investigate the effect of CBM on NP and spasticity in patients with MS or SCI. We performed a randomized, double-blinded, placebo-controlled trial in Denmark. Patients aged ≥18 years with NP (intensity >3, ≤9 on a numerical rating scale (NRS0-10) and/or spasticity (>3 on NRS0-10) were randomized to treatment consisting of either delta-9-tetrahydrocannabinol (THC), cannabidiol (CBD), a combination of THC&CBD in maximum doses of 22.5 mg, 45 mg and 22.5/45 mg per day, respectively, or placebo. A baseline registration was performed before randomization. Treatment duration was six weeks followed by a one-week phaseout. Primary endpoints were the intensity of patient-reported NP and/or spasticity. Between February 2019 and December 2021, 134 patients were randomized (MS *n* = 119, SCI *n* = 15), where 32 were assigned to THC, 31 to CBD, 31 to THC&CBD, and 40 to placebo. No significant difference was found for: mean pain intensity (THC 0.42 (−0.54–1.38), CBD 0.45 (−0.47–1.38) and THC&CBD 0.16 (−0.75–1.08)), mean spasticity intensity (THC 0.24 (−0.67–1.45), CBD 0.46 (−0.74–1.65), and THC&CBD 0.10 (−1.18–1.39), secondary outcomes (patient global impression of change and quality of life), or any tertiary outcomes. We aimed to include 448 patients in the trial; however, due to COVID-19 and recruitment challenges, fewer were included. Nevertheless, in this four-arm parallel trial, no effect was found between placebo and active treatment with THC or CBD alone or in combination on NP or spasticity in patients with either MS or SCI. The trial was registered with the EU Clinical Trials Register EudraCT (2018-002315-98).

## 1. Introduction

Central neuropathic pain (NP) and spasticity are common consequences of central nervous system diseases with a major impact on quality of life [1,2]. In multiple sclerosis (MS), inflammation in the central nervous system (CNS) leads to demyelination and neurodegeneration which can cause permanent disability [3]. Spinal cord injury (SCI) is a spinal cord lesion due to trauma or disease. Both MS and SCI may lead to significant problems with NP and spasticity [1,3,4].

Traditional analgesic and antispastic medicine often have an insufficient effect on NP and spasticity. In Denmark, the International Association for the Study of Pain (IASP) recommendations for the treatment of NP are followed with first-line treatments being gapapentin, pregabalin, serotonin-noradrenalin reuptake inhibitors (SNRI), and tricyclic antidepressants (TCA). In recent years, cannabis and cannabis-based medicine (CBM) have been suggested for medicinal purposes for a wide range of symptoms, including NP and spasticity. However, the effect on spasticity is limited [5,6,7] and the recent IASP presidential task force on cannabis and cannabinoid algesia concluded that “due to lack of high-quality clinical evidence, the use of cannabis/CBM is not currently endorsed for pain relief” [8]. Nevertheless, patients request CBM and are sometimes under the impression that it is more safe than conventional medicine [9]. The two most well-known cannabinoids are the psychoactive delta-9-tetrahydrocannabinol (THC) and the non-psychoactive cannabidiol (CBD) [10]. The present study aimed to investigate the effect of THC and CBD (alone and combined) in capsule formulation on NP and spasticity in patients with MS or SCI. Pain and spasticity were evaluated separately for patients fulfilling the pain and spasticity criteria, respectively. A secondary aim was to assess the adverse events (AE) of THC and CBD.

## 2. Results

### 2.1. Screenings and Dropout Characteristics

**Figure 1:**
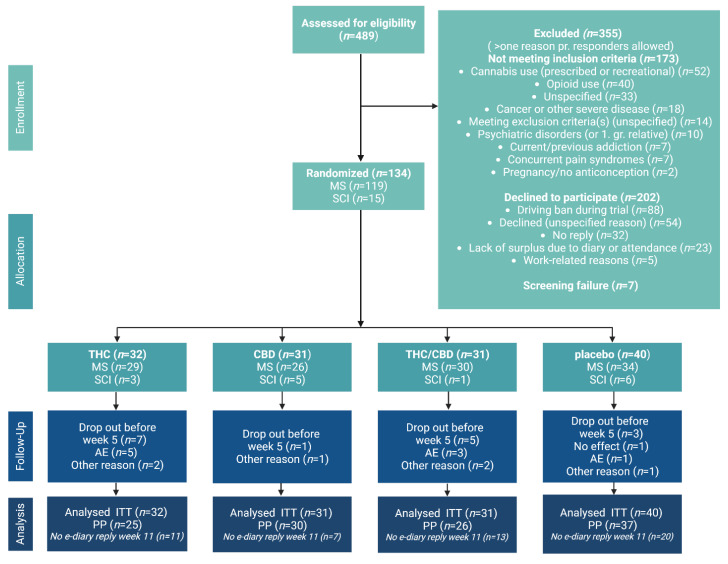
CONSORT flow diagram. Flow diagram for the trial. MS: multiple sclerosis, SCI: spinal cord injury, THC: delta-9-tetrahydrocannabinol, CBD: cannabidiol, THC&CBD: delta-9-tetrahydrocannabinol and cannabidiol. AE: adverse event, ITT: intention to treat, PP: per protocol.

Of the 489 patients assessed for eligibility, 355 (72.6%) did not meet the inclusion/exclusion criteria (*n* = 173, mostly due to ongoing use of cannabinoid/cannabis/CBM/opioids), or declined to participate (*n* = 202, mainly due to the driving ban during the treatment phase (*n* = 88)) (Figure 1). No inclusion or exclusion criteria were changed during the study period.

### 2.2. Patients

In total, 134 patients (27.4% of all screened) were randomized (119 patients with MS (88.9%) and 15 patients with SCI (11.2%)) to one of the four allocation groups, placebo (*n* = 40), THC (*n* = 32), CBD (*n* = 31), or THC&CBD (*n* = 31). The inclusion criteria for NP were fulfilled in 114 patients (85.1%) and for spasticity in 100 patients (74.6%). Eighty patients (59.7%) had both NP and spasticity (73.3% of patients with SCI and 58% of patients with MS, respectively). Dropouts are listed in the flowchart (see Figure 1). Baseline demographics are listed in Table 1. No major differences were seen between the four allocation groups for any of the baseline demographics. Baseline characteristics from questionnaires, tests, and expectations of relief are listed in Table 2. Due to recruitment difficulties and the COVID-19 pandemic, the aimed sample size was not reached at the planned end of the study. We observed no progression in disability from baseline to endpoint evaluated by EDSS or AIS. Many patients were on ongoing treatment with analgesics for NP or spasticity (Table 1) or had tried traditional approved medicine without sufficient effect.

### 2.3. Primary Outcomes

#### Pain

All four groups had a significant decrease in mean intensity of NP from baseline to endpoint in the ITT analysis (placebo −1.8 (SD 1.8), THC −1.4 (SD 2.0), CBD −1.4 (SD 1.6), and THC&CBD −1.6 (SD 1.8). When comparing the three active treatment groups with placebo, no significant difference in mean pain intensity was found in THC 0.42 (−0.54–1.38), CBD 0.45 (−0.47–1.38), and THC&CBD 0.16 (−0.75–1.08) (one-way ANOVA *p* = 0.74) (Table 3). No interaction was observed between THC and CBD, and no effect was seen on single drugs using two-way ANOVA (Table 3).

### 2.4. Spasticity

All four allocation groups had a significant decrease in mean intensity of spasticity from baseline to endpoint in the ITT analysis (placebo −1.7 (SD2.0), THC −1.5 (SD2.0), CBD −1.3 (SD1.9), and THC&CBD −1.6 (SD2.7). A comparison of the three active treatment groups compared to the placebo group showed no significant difference in spasticity reduction THC 0.24 (−0.67–1.45), CBD 0.46 (−0.74–1.65), and THC&CBD 0.10 (−1.18–1.39) (one-way ANOVA, *p* = 0.89) (Table 3). There was no interaction between THC and CBD and no effect of single drugs using two-way ANOVA (Table 3).

### 2.5. Supplement Analysis and Results

A BOCF analysis was made for the primary effect parameters (pain and spasticity), which did not change the results significantly (Table 3). Also, an analysis of only the MS population was conducted, which did not change the result significantly (not presented). An analysis of week-by-week mean NP and spasticity for the PP population can be seen in the (Supplementary Figure S1). During follow-up, the mean pain and spasticity intensities approached the baseline values. For patients fulfilling both inclusion criteria, high consistency was observed between changes in pain and spasticity for all groups (Figure S2).

### 2.6. Secondary Outcomes

**Figure 2:**
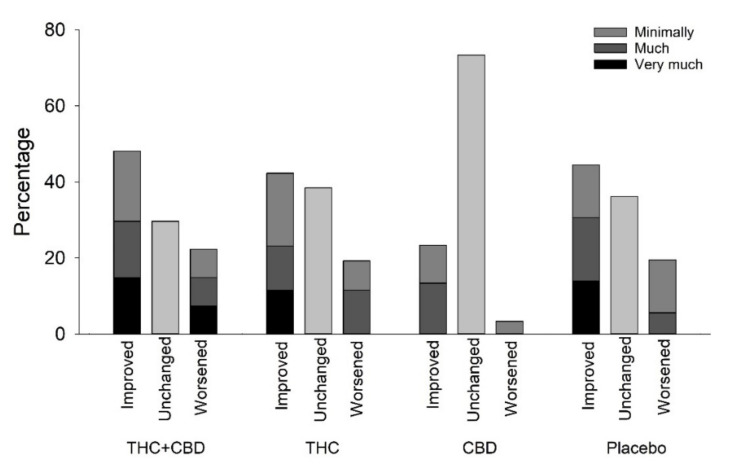
Patient Global Impression of Change. Patient Global Impression of Change (Kruskal–Wallis *p*-value = 0.82). THC: delta-9-tetrahydrocannabinol, CBD: cannabidiol, THC&CBD: delta-9-tetrahydrocannabinol and cannabidiol.

No significant differences were seen in PGIC (Figure 2) or quality of life between the four allocation groups assessed by the EuroQol Group 5Q-5D-5L questionnaire (Table 4). 

### 2.7. Other Outcomes

No significant differences between the four allocation groups were found for any of the tertiary pain or spasticity outcomes (Table 3). No significant difference was found between the four allocation groups for any of the remaining outcomes or use of escape medication (*p* = 0.18) (Table 4).

### 2.8. Daily Dose and Blinding Assessment

The median daily dose in the stable phase was lower in the THC&CBD group than in the other allocation groups (7 vs. 9 capsules a day) (*p* < 0.01). More patients guessed that they were in active treatment in the THC&CBD group (64.3%) and THC group (53.8%) than in the placebo group (44.4%) and CBD group (16.7%) (*p* = 0.012) (Table 4). To maintain the blinding no drug test was performed after randomization. In the sub-study evaluating the pharmacokinetics, no cannabinoids were detected in the placebo group, no THC was detected in the CBD group, and no CBD was detected in the THC group (published separately). This indicates that the use of supplemental/recreational cannabis during the trial was probably minimal.

### 2.9. Expectation

All four groups had high expectations of the effect of the active drugs (median of 8.0 on the 0–10 NRS score). No difference was observed in expectations between the groups. No statistically significant correlation was found between expectations and change in pain or spasticity. 

### 2.10. Adverse Events and Safety

AEs were reported by 119 patients (88.8%). Most patients reported more than one AE, and in total 537 reports were evaluated. After evaluation, a possible or certain relation between AE and the study drug was found in 97 patients (72.4%). Each AE was included only once per patient.

The THC and the THC&CBD groups most frequently reported AE (Table 5, Table S1 and Figure S3). Overall, three serious adverse reactions/suspected unexpected serious adverse reactions (SAR/SUSARS) that led to hospitalization were reported during the study. Two were in the THC group (dizziness/nausea/headache and dizziness/fall), and one was in the CBD group (gallstone attack in a patient with known gallstone and history of gallstone attacks). Four serious adverse events (SAE) were reported but evaluated as not related to the study mediation. Two in the THC group and two in the placebo group. They were all due to infections (fever, pneumonia, UTI, or decubitus). No changes in the evaluated blood samples were observed during the trial. In the THC group at the endpoint, one had ectopic extrasystoles that disappeared after the study ended, and two had sinus bradycardia that was not present at inclusion. No patients were admitted to the hospital with COVID-19 during the study and no COVID-19 was reported in the AE registration.

## 3. Discussion

In this four-armed, double-blinded, randomized, placebo-controlled study investigating oral capsule THC (max. dose 22.5 mg/day) and CBD (max. dose 45 mg/day), no difference was found between THC, CBD, THC&CBD, and placebo in relieving pain and spasticity intensity in patients with MS or SCI. Also, no differences in the secondary or tertiary outcomes were found. AEs were more often reported with THC alone or in combination with CBD. In the general population, CBM has been praised for alleviating various symptoms, and interest in/request for legal medical cannabis or CBM has been considerable. Among Danish patients with MS, the four most frequently stated positive effects of (and reasons to use) medical cannabis were ease of pain, ease of spasticity, better sleep, and “feeling relaxed”. In the same report, more than 60% reported “no negative effects” when using cannabis as medicine, and only a few percent reported AEs [9]. In our study, no effect was found on NP, spasticity, or sleep. Our findings are in concordance with the recent recommendation from the IASP presidential task force and the results of a systematic review by Fisher et al. that found limited evidence for the use of cannabinoids in pain management, and a higher rate of AEs in THC (or THC-containing) treatment groups [8,11]. Patients in the THC and THC&CBD groups encountered significantly more AEs than the placebo group. The CBD group, on the other hand, did not differ from the placebo group. We used synthetic CBD to ensure that the isolated cannabinoid was investigated and because no medical-grade plant-based CBD was available at the time of trial approval [12]. The psychoactive AEs, such as hallucinations, anxiety, and nervousness were not frequently reported in any of the groups, and no statistically significant difference was seen between the groups on these parameters. Previously, it has been suggested that CBD limits the (psychiatric) AEs of THC [13]. However, our results did not support this as the THC&CBD group reported the most “other” AEs. Interestingly, the THC&CBD group was titrated to a significantly lower dose than the other groups. AEs were mostly mild to moderate. The doses of cannabinoids chosen for this study was reflecting the available orally formulated cannabinoids in the Danish pilot scheme for medical cannabis, the consensus recommendations from cannabis-prescribing doctors, and what has been observed from retrospective reports [14,15,16]. In recent years, the use of isolated CBD or CBM with a high CBD:THC ratio has markedly increased. Only one recent placebo-controlled study by Vela et al. (2022). assessed CBD (doses 20–30 mg/day) for pain (hand osteoarthritis and psoriatic arthritis), and it failed to find any effect [17]. Another recent Danish trial by Zubcevic et al. (2023). evaluating CBM on peripheral NP in a design very similar to the present study (outcome measure and number of patients) did not find an effect of CBD, THC, or the combination of THC and CBD on peripheral neuropathic pain [18]. Furthermore, no effect of Dronabinol (synthetic THC) was found on central pain in the study by Schimrigk et al. (2017) [19]. The results from these studies evaluating CBM on pain are in accordance with the findings in this present study. Earlier randomized studies found a small effect of the combination of THC and CBD (nabiximols) on the central end peripheral NP (Rog et al. (2005), Nurmikko et al. (2007)) [20,21]. However, in a larger 14 weeks RCT by Langford et al. (2012) also investigating nabiximols the primary endpoint of a 30% pain reduction was not met [22]. To our knowledge, no previous studies have investigated the effects of isolated CBD on spasticity [23] and, at this point, CBD (high dose) has shown effects in severe childhood epilepsy only [24]. The psychoactive THC has been more widely investigated than CBD, both in combination formulation (nabiximols) and solitarily (both synthetic and natural) [25]. Studies, however, are either small, severely biased, or have shown divergent results. The use of psychoactive THC for pain is currently not endorsed [11,26]. A small positive effect of nabiximols on spasticity in MS has been reported [27,28,29,30]. In a recent Cochrane review (2022), the authors conclude that nabiximols probably reduce the severity of spasticity in patients with MS (short-term). In this review, an unpublished study (oral THC/CBD) with unknown status was included (NCT03005119) [26]. The placebo response in our study was considerable. Since we did not include a non-treated control group, we cannot tell how much of the placebo response is due to a placebo effect. Nevertheless, the placebo response in trials investigating CBM is substantial as reviewed by Gedin et al. [31]. The design as a parallel study and a 75% chance of receiving active treatment may also potentiate the placebo response in the present study. We tried to keep visits and information balanced so that safety and endpoint assessments were obtained without facilitating the placebo response. Also, patients were made clear of the chance of being given a placebo and that the effect of CBM was indefinite. Since the pain and spasticity intensity on average returned to baseline levels after the end of treatment, it suggests that the placebo response was not only explained by regression to the mean. CBM has received substantial media attention and this may explain our findings of very high expectations of both pain and spasticity relief [31]. Even so, we found no significant correlation between expectation and mean pain or spasticity difference. As the study medication was used as an add-on to patients ongoing treatment with analgesics or antispastics and the aim of the study was to compare different cannabinoids with placebo, no “positive” control group (using first-line treatments) was included. This, however, could be an interesting topic for future studies.

To our knowledge, this is the first study that investigates the oral formulation of CBD and the interaction with THC on central NP and spasticity in a double-blinded RCT design. Furthermore, the study is one of the largest studies evaluating the effects of CBM on central NP in a four-arm parallel design where THC and CBD are investigated both separately and together. We used patient-reported outcomes (NRS0-10) and e-diaries along with several other outcomes such as PGIC, 30%, and 50% responders, and objective outcomes (e.g., cMAS). Both expectations of the effect of CBM and “blinding assessment” were assessed, and pain and spasticity were evaluated during phaseout and follow-up (e-diary). We used oral formulation capsules to ensure an exact dose, and we allowed for individual dose titration. AEs were comprehensively evaluated.

Our study has limitations for consideration. First, we did not reach the planned number of patients. This was due to the COVID-19 pandemic and the reluctance of patients to participate due to the driving ban during the study, ongoing use of opioids or cannabis/CBM, and other reasons. However, our power calculations were probably too conservative as a higher number of the MS patients had both NP and spasticity, the actual SD of mean differences were lower than expected, and we found no interaction between THC and CBD treatment. Despite a numerically better effect of a placebo than each active drug, considering the confidence interval of estimated effect sizes, we cannot exclude a small effect of the treatments. However, except for the THC&CBD treatment for spasticity, where the confidence interval ranged from 1.18 in favor of treatment to 1.39 in favor of placebo, the maximal possible effects of the treatment were below 0.75 on an NRS0-10, which suggests a non-clinically relevant/minimally important change in NRS [32]. Clinical studies were paused during the COVID-19 lockdown and when inclusion was again possible, numerous restrictions in the society were still present, which we hypothesized affected the willingness to participate in a clinical trial. Especially since patients with MS may be at higher risk of infections due to their immunomodulation therapy.

Other limitations included the risk of unblinding either due to the effects/side effects or due to patient intentional unblinding for example using THC self-test [33], we did not contact the patient to re-evaluate the blinding assessment. In contrast, it was a strength of the study that blinding was assessed at the endpoint, and it was unanticipated that 63.3% in the CBD group guessed that they were receiving a placebo (compared with 44.4% in the placebo group).

We cannot exclude that a higher dose of CBD, which was well tolerated, could have yielded other results. In addition, due to first-pass metabolism in the liver, oral formulation cannabinoids have low bioavailability, and other formulations might have had different effects. We did a baseline drug test at inclusion, but we cannot rule out the small risk of “cheating” with other kinds of cannabis during the study (in the sub-study however, it did not seem to be the case from the evaluated blood samples). We did not register if patients had ever used cannabis. However, we assessed the expectation of treatment, which did not predict the outcome. Current cannabis users were excluded, which might have resulted in a lower number of responders in this trial with an underestimate of the efficacy of CBM. Patients were allowed to continue stable analgesics or antispastic medication throughout the treatment phase, with the risk of a lower observed effect. However, all included patients had persistent pain and/or spasticity intensity >3 at inclusion; moreover, in all treatment groups, a reduction in pain/spasticity was seen during the study, but with no difference between active treatments and placebo. Since cannabinoids are primarily metabolized in the liver via the CYP450-system, there is a potential risk of drug-drug interaction with other therapies metabolized mainly via CYP3A4, CYP2C9, and CYP2D6 (including traditional first-line treatment for NP: TCA and SNRI). Opioids were not allowed due to the risk of drug-drug interaction and because opioids have only limited eligibility in the treatment of NP in Danish guidelines. Yet, little is still known about the significance of potential drug-drug interaction, and further discussion of drug-drug interaction is beyond the scope of this trial. It might have been difficult for patients to distinguish between NP and painful spasticity despite being introduced to this at inclusion, and our results suggested that the outcomes were not completely independent for patients who fulfilled both inclusion criteria. Finally, the treatment duration was seven weeks; this means that we cannot provide information on long-term AEs and risks, but we considered the study period to be long enough to evaluate an effect in a steady state. Despite limitations, this RCT contributes valuable information on the effects of CBM on NP and spasticity in patients with MS and SCI. In addition, it is, to our knowledge, the first clinical trial to evaluate isolated CBD on central NP and spasticity.

## 4. Materials and Methods

### 4.1. Study Design and Settings

The study was designed as a national multicenter, randomized, double-blinded, placebo-controlled (RCT) study, coordinated by a steering committee, with investigators at ten MS and two SCI outpatient clinics in Denmark. The study was conducted according to the Helsinki Declaration Guidelines and approved by the Danish Medicine Agency (2018071161), the Science Ethics Committee (VEK1-10-72-291-18), the Data Protection Agency (via the Central Denmark Region’s internal notification 1-16-02-582-18), and it was registered with the EU Clinical Trials Register EudraCT (2018-002315-98). The study was an investigator-initiated trial that followed the Good Clinical Practice (GCP) guidelines and was monitored by GCP units from Aarhus, Odense, and Copenhagen Universities, Denmark. REDCap^®^ hosted by Aarhus University was used for data storage and e-diary [34,35]. The study protocol and statistical analysis plan were published previously [12].

### 4.2. Study Patients

Patients with MS or SCI were included between February 2019 and December 2021. Inclusion was paused twice: first, due to additional documentation from the laboratory that controlled the study medicine (please note that the documentation was not related to the study medicine) (paused from November 2019–February 2020) and, second, due to the COVID-19 lockdown (from March 2020–May 2020, during the remaining pandemic, the inclusion rate was lowered). Inclusion criteria were: age ≥ 18 years, definite or probable NP (according to the NP grading system [36]) for more than three months with mean intensity at baseline (one-week registration) >3 and ≤9 on a numerical rating scale (NRS0-10) and/or presence of spasticity for more than three months with a mean intensity at baseline >3 (NRS0-10). Stable disease was required (for MS no change in disease-modifying treatment during the previous three months and no relapse in the past month). Informed consent was obtained for all included patients. The exclusion criteria included competitive pain diseases, opioid treatment, active use of cannabis (or cannabis-containing products) within the past 3 months (both recreational and medical), severe co-morbidities (active cancer, epilepsy, heart, kidney, or liver disease), risk of suicide (assessed by the Columbia Suicide Severity scale [37]), previous psychiatric disease in patient or first-degree relatives, pregnancy, breastfeeding, planned surgery, or travels abroad during the intervention period. Standard blood samples, blood pressure, and ECG were collected at visits 1, 3, and 4 (Figure S4). Patients were not allowed to drive motorized vehicles or use cutting tools during the treatment phase. Blood pressure was evaluated using routine methods. Clearblue^®^ pregnancy test (urine, fertile women at visits 2 and 3), and Ferle NanoSticka THC 20 ng/mL^®^ drug test (urine, all patients at visit 2) were used for screening. Height and weight were patient-reported if known or measured using standard methods (visits 1 and 4). A bedside neurological examination was performed by a neurologist at inclusion to ensure the probability of pain being neuropathic and to grade the spasticity if present. Anticonception should be used during and three months after treatment. Concomitant therapies and medication were required to be maintained at a stable dose, and paracetamol was allowed as escape medicine.

### 4.3. Study Visits

After inclusion (visit 1), a seven-day baseline period was followed by randomization (visit 2). Next was a three-week titration period with three consultations per telephone, the last of which could be combined with control visit 3. Then followed three weeks of stable phase (same dose each day). The endpoint (visit 4) was performed before a one-week phaseout period terminated by visit 5 (Supplementary Figure S4). A follow-up phone consultation was performed 4 weeks after the last dose of study medication. From inclusion to phaseout and again at follow-up, the patients completed an electronic diary (e-diary).

Supplementary phone consultations were performed when needed during the trial. If the patients were not able to keep an e-diary, a printed version was offered. In case of discontinuation/withdrawals, patients were asked to complete visit 4 and the e-diary. If this was not possible or if they wished to withdraw entirely from the project, a telephone consultation was undertaken two weeks after withdrawal.

### 4.4. Randomization and Blinding

Eligible patients were randomized after the seven-day baseline period. Patients were randomized to treatment with either THC, CBD, THC&CBD, or placebo in a 1:1:1:1 ratio. Glostrup Pharmacy performed the computer-generated randomization list. Block randomization (block sizes 10–15) was used at the three largest sites (Aarhus, Viborg, and Copenhagen). The treatment allocation list was revealed on 1 March 2022 (after the last patient’s last visit and closure of the database). The AEs were evaluated before treatment allocation was revealed. No drug test was performed after randomization.

### 4.5. Intervention

The study medicine was approved by the Danish Medicine Agency and produced at the “Good Manufacturing Practice”-certified Glostrup Pharmacy, Denmark, and consisted of capsules (hard gelatin, white size 1, identical regarding look, smell, taste, and color) containing either Dronabinol (natural THC) 2.5 mg, Cannabidiol (synthetic CBD) 5 mg, Dronabinol (natural THC) 2.5 mg, and cannabidiol (synthetic CBD) 5 mg or placebo (hard fat and no active components). CBD was synthetic as no natural extract of CBD was approved by the Danish Medicine Agency when the trial was initiated. The maximum daily dose was nine capsules (divided into three daily doses, THC max dose = 22.5 mg, CBD max dose = 45 mg). The daily dose was titrated for three weeks using a standard escalation schedule. If patients experienced an effect at a lower dose or AEs when escalating, individual lower doses were accepted (min. one capsule/day). The dose chosen was equal to the approved/available orally formulated CBM in the Danish pilot program for CBM [14]. The study medicine was used as an add-on to patients’ ongoing therapy and medicine (including antispastics and analgesics). Patients were instructed to take the study medication along with a meal to enhance the drug’s bioavailability [12]. The manufacturer had no clinical involvement in the project.

### 4.6. Outcome Measures

The study had two primary outcomes: For patients fulfilling the pain criteria, the primary outcome was a change in pain intensity. For patients fulfilling the spasticity criteria, the primary outcome was a change in spasticity intensity. Change in pain intensity was defined as the difference in mean pain intensity from the baseline week to the last seven days of treatment (before phaseout) recorded in the e-diary on an NRS0-10 (0 being no pain, 10 being the worst imaginable pain) for patients who fulfilled the criteria of NP > 3 and ≤9 at baseline. Change in spasticity intensity was defined as the difference in self-reported intensity of spasticity (from mean intensity during baseline week to the last seven days of treatment (before phaseout) on an NRS0-10 (0 being no spasticity, 10 being the worst imaginable spasticity) for patients who fulfilled the criterion of spasticity >3 at baseline. If patients fulfilled both criteria at randomization, both outcomes were evaluated. Secondary outcomes were the Patient Global Impression of Change (PGIC) (“What is your impression of change (if changed) in your general condition/well-being from the week before treatment to the last week (of treatment in steady state)?” (from very much worsened to very much improved)), and quality of life (EuroQol Group 5Q-5D-5L) [38]. Tertiary pain outcomes (for patients fulfilling the pain inclusion criteria) were: numbers of responders with 50% pain reduction (30% pain reduction was reported in addition), pain relief (NRS1-10 e-diary, endpoint), and NP descriptors measured on the Neuropathic Pain Symptom Inventory (NPSI) [39]. Tertiary spasticity outcomes were the (combined) modified Ashworth Scale (cMAS) [40] (evaluating left and right side: elbow, wrist, fingers, hip, knee, and ankle joints (range 0–48)), the number of patients with 50% spasticity reduction (30% spasticity reduction was reported in addition), and spasticity relief (NRS1-10 e-diary, endpoint). Other tertiary outcomes included impact on sleep (“Has your night’s sleep last night been influenced by your pain and/or your spasticity?” (NRS0-10, 0 = no influence, 10 = worst possible influence), e-diary, daily) and sleep quality, anxiety, and depression (patient-reported outcome measurement information system (PROMIS Neuro-QOL) short form 6a in the Danish language, e-diary at baseline, and weeks five and six) [41]. At baseline and endpoint, four clinical tests were performed. The following cognitive tests were applied in the study: The paced auditory serial addition test (PASAT, 3-s rate) [42] and the verbal Symbol Digit Modalities Test (SDMT) [43] were used as processing speed tests, trail-making test A was applied as a visuomotor function test, and the trail-making B test was used to measure executive functioning [44]. The 9-hole peg test (9HPT) was used to evaluate hand coordination [45]. The tests also served as safety measures. The use of escape medication was reported in the e-diary (daily). Daily dose at endpoint and dropouts were registered. The Expanded Disability Status Scale (EDSS) for MS was evaluated at baseline and endpoint to check for disease progression in MS. For SCI, investigators evaluated at the endpoint if any changes had occurred in the American Spinal Injury Association Impairment Scale (AIS).

As a predictor, the patients’ “expectation of relief” (for pain and spasticity, respectively) at baseline (“on a 0–10 scale, how well do you expect CBM affects your pain/spasticity (NRS0-10, 0 = no effect, 10 = removes pain/spasticity completely) was evaluated. At visit 4, the patients indicated what kind of treatment (active vs. placebo) they thought they had received. All AEs (weekly in e-diary and at visits/phone consultation) (list: mouth dryness, headache, depressive thoughts, nightmare, euphoria, dizziness, tinnitus, anxiety, hallucinations, fatigue/drowsiness, palpitations, flushing of the face, stomachache, nausea, diarrhea, muscle pain, visual disturbances, and other (open question)) were collected. All AE questionnaires were evaluated by the sponsor site and allocated to “previous reported and unrelated”, “unrelated”, “possible or certainly related”, and “cannot be assessed”. An AE was included in the inventory if it was rated “possible” or “certainly related” once during the trial. If there was any doubt that the AE was related, it was reported as “possibly” related. One AE could be reported multiple times by each patient but was included only once per patient. A sub-study regarding the pharmacodynamics and pharmacokinetics of cannabinoids and metabolites was performed and described in the protocol article [13]. Results will be published separately.

### 4.7. Clinical Laboratory Tests

The blood sample panel included plasma sodium, potassium, creatinine, estimated glomerular filtration rate, alanine aminotransferase, urea, alkaline phosphatase, bilirubin, total cholesterol, low-density lipoprotein cholesterol, high-density lipoprotein cholesterol, triglyceride, leukocytes, and hemoglobin. In the case of blood samples beyond reference values, this was noted, evaluated, and repeated/acted on continuously.

### 4.8. Power and Sample Size Considerations

Considering a combined standard deviation of 3.5, an effect of 1 point mean difference (in pain or spasticity) on the NRS0-10 scale, a power of 80%, and uneven distribution in pain and spasticity between the two patient groups (MS 40% and SCI 70%), we aimed to include 112 patients in each of the four groups (THC, CBD, THC&CBD, and placebo) [EudraCT 2018-002315-98)].

### 4.9. Statistical Analysis

Baseline characteristics/variables were described for all patients with count and percentages (and range) for discrete variables, and with mean and standard deviation (SD) or median and interquartile range (IQR) (25–75 percentiles) for continuous variables according to a sample distribution. For the primary outcomes (pain and/or spasticity (NRS0-10)), the mean value from the baseline week was compared with the mean value of the last seven days of active treatment (endpoint) (from the e-diary). The analysis was based on an intention-to-treat (ITT) principle with the last observation carried forward (LOCF). Patients were included in ITT if they had taken at least one capsule of the study medication. Patients who completed at least four weeks of treatments (=steady state) were considered case-completed and included in the “per-protocol” (PP) analysis. For the primary outcomes, an additional “baseline observation carried forward” (BOCF) analysis was performed for patients not fulfilling the PP criteria. The normality of the variable sample distribution was visualized using QQ-plot, and a scatterplot was used to examine the relationship between the allocation groups. The predefined analysis was the one-way analysis of variance (one-way ANOVA) for normally distributed data or Kruskal–Wallis, if not normally distributed data. In addition, to better assess the effect of each of the cannabinoids alone (THC and CBD) and their interaction, a two-way ANOVA model for the primary (and secondary) outcomes was used. A paired *t*-test was performed to compare the baseline and the last seven days of active treatment (endpoint) within each allocation group. A MIXED model with allocation group, mean pain difference, and interaction of them as fixed effects and patient-indicator variables as a random effect with unstructured covariance was fitted for the week-by-week mean pain and spasticity analysis of the PP population. The MIXED model was also applied to evaluate the use of escape medication week-by-week. For nominal data, differences between groups were analyzed using Fischer’s exact test. Safety outcomes included registration of all possible AEs during the trial (reported at visits or weekly in e-diary) and were reported as the percentage of each group (the AE was registered the first time it occurred). A Chi-square test for each of the active groups compared with the placebo was performed for the 18 predefined AEs and “others”. A *p*-value < 0.05 was considered statistically significant. A Spearman correlation was calculated to assess the relationship between expectation and pain/spasticity difference. Data management and analysis were performed using STATA version 17.0 (StataCorp. College Station, TX, USA).

## 5. Conclusions

No difference was found between placebo and active treatment with oral capsule THC or CBD alone or in combination on NP or spasticity in patients with MS or SCI. No difference was found in secondary and tertiary outcomes besides more AEs in the THC-containing treatment groups.

## Figures and Tables

**Table 1:** Demographics and baseline parameters.

	Placebo	THC	CBD	THC&CBD
Total No.	40	32	31	31
Diagnosis				
MS, *n* (%)	34 (85.0)	29 (90.6)	26 (83.9)	30 (96.8)
SCI, *n* (%)	6 (15)	3 (9.4)	5 (16.1)	1 (3.2)
Sex				
Female (*n* = 99), *n* (%)	28 (70.0)	21 (65.5)	22 (71.0)	28 (90.3)
Male (*n* = 35), *n* (%)	12 (30.0)	11 (34.4)	9 (29.0)	3 (9.7)
Age at randomization				
Years, mean (SD) range	52.2 (10.4) 21–70	54.1 (10.4) 30–69	53.0 (9.8) 30–73	51.1 (12.7) 30–84
Time since diagnosis (MS/SCI)				
Years, mean (SD) range	13.3 (7.9) 1–32	14.8 (11.8) 0–38	13.7 (8.2) 1–31	11.5 (7.3) 0–30
BMI kg/m^2^, mean (SD)	25.5 (5.1)	26.4 (6.0)	26.8 (5.1)	26.0 (5.6)
Patients with pain >3, ≤9 at baseline,				
*n* (%) (total *n* = 114) NRS0-10	35 (87.5)	24 (75.0)	27 (87.1)	28 (90.3)
Pain at baseline mean (SD)	6.0 (1.3)	6.6 (1.7)	6.0 (1.4)	5.7 (1.4)
Patients with spasticity >3 on NRS0-10 at baseline, *n* (%) (total *n* = 100) Spasticity at baseline mean (SD)	29 (72.5) 5.9 (1.4)	25 (78.1) 5.7 (1.6)	26 (83.9) 5.9 (1.4)	20 (64.5) 5.7 (1.5)
EDSS (MS) median (IQR)	4.5 (3.0–6.0)	6.0 (3.5–6.0)	6.0 (5.0–6.5)	4.5 (3.0–6.0)
AIS (SCI)				
A	1	-	2	-
B	1	2	-	-
C	2	-	-	-
D	2	1	3	1
Level of lesion				
Cervical	3	1	1	-
Thoracal	3	2	3	1
Lumbar	-	-	1	-
SCI type				
Traumatic	1	1	2	-
Non-traumatic	2	2	1	1
Other/Unknown	3	-	2	-
Other medication				
Gabapentin/pregabalin	11	10	7	9
SNRI	2	0	4	3
TCA	2	4	1	1
Other pain treatment	24	23	18	20
Antispastics	17	14	12	14

THC: delta-9-tetrahydrocannabinol, CBD: cannabidiol, THC&CBD: delta-9-tetrahydrocannabinol and cannabidiol, MS: multiple sclerosis, SCI: spinal cord injury, BMI: Body Mass Index, EDSS: Expanded Disability Status Scale (MS) (0–10, the scoring is based on examination by a neurologist. 0 = no symptoms, 1–4.5 = patients able to walk without any aid, 5–9.5 = walking impairment or confined to bed, 10 = death due to MS). IQR: interquartile range (25–75%), AIS: American Spinal Injury Association (ASIA) Impairment Scale (A = Complete Injury. No sensory or motor function is preserved in the sacral segments S4–S5, B = Incomplete. Sensory but not motor function is preserved below the neurological level and includes the sacral segments S4–S5, C = Incomplete. Motor function is preserved below the neurological level, and more than half of the key muscles below the neurological level have a muscle grade of less than 3, D = Incomplete. Motor function is preserved below the neurological level, and at least half of key muscles below the neurological level have a muscle grade greater than or equal to 3. Oher medication = patient medication at time of inclusion, only medication for pain/spasticity is reported. SNRI = serotonin-noradrenalin reuptake inhibitor, TCA: tricyclic antidepressants. Other pain treatment includes (one or a combination of) paracetamol, acetylsalicylic acid, carbamazepine, and non-steroid anti-inflammatory drugs. Antispastics include baclofen, tizanidine, chlorzoxazone, and botulinum toxin.

**Table 2:** Baseline characteristics (questionnaires and tests).

	Placebo	THC	CBD	THC&CBD
Total *n*	40	32	31	31
Impact on sleep (NRS0-10)				
Baseline, mean (SD)	4.7 (2.2)	4.8 (2.4)	4.9 (2.0)	3.9 (2.3)
NPSI (*n* = 101 *)	(*n* = 31)	(*n* = 20)	(*n* = 26)	(*n* = 24)
The total sum, median (IQR)	2.6 (1.6–3.9)	2.5 (1.7–4.8)	3.5 (2.2–4.7)	2.7 (2.0–4.9)
Burning pain, median (IQR)	5.0 (2.0–6.0)	5.0 (0.5–7.5)	5.0 (0.0–6.0)	5.0 (0.0–7.0)
Pressing pain, median (IQR)	3.0 (0.0–5.0)	3.5 (1.5–7.0)	4.3 (2.0–7.0)	3.8 (1.8–5.5)
Pins and needles/tingling, median (IQR)	2.5 (0.0–5.0)	2.5 (0.0–4.0)	3.0 (2.0–6.5)	2.5 (0.0–5.8)
Evoked pain, median (IQR)	1.7 (0.0–3.0)	1.8 (0.0–3.2)	3.0 (0.0–5.0)	2.2 (0.0–4.8)
Paresthesia/dysesthesia, median (IQR)	2.5 (0.0–5.0)	2.5 (0.0–6.3)	3.5 (2.0–5.0)	2.5 (0.5–5.5)
The expectation of relief NRS0-10 ^§^				
Pain * (*n* = 111), median (IQR)	8.0 (7.0–10.0)	8.0 (5.5–9.0)	8.0 (5.0–10.0)	8.0 (7.0–10.0)
Spasticity ** (*n* = 99), median (IQR)	8.0 (6.0–10.0)	8.0 (6.0–8.0)	8.0 (6.0–10.0)	8.0 (7.0–9.0)
Quality of life 5Q-5L-5D				
Index, mean (SD)	0.6 (0.2)	0.6 (0.2)	0.5 (0.2)	0.6 (0.2)
9-Hole Peg Test (s)	(*n* = 36/34)	(*n* = 28/29)	(*n* = 29/28)	(*n* = 29/27)
Dom hand, median (IQR)	23.5 (20.0–29.1)	25.3 (20.8–30.8)	24.0 (21.0–29.0)	23.1 (19.0–27.8)
Non-dom hand, median (IQR)	24.6 (20.9–31.0)	28.6 (23.1–42.0)	23.9 (22.1–29.7)	21.9 (18.8–28.8)
PASAT (0–60)	(*n* = 32)	(*n* = 28)	(*n* = 28)	(*n* = 27)
Baseline, mean (SD)	39.3 (9.0)	38.6 (14.9)	38.2 (14.8)	40.4 (15.3)
TMT A (s)	(*n* = 36)	(*n* = 31)	(*n* = 31)	(*n* = 31)
Baseline mean (SD)	45.8 (37.3)	45.3 (33.3)	46.4 (26.1)	42.9 (31.4)
TMT B (s)	(*n* = 36)	(*n* = 31)	(*n* = 31)	(*n* = 29)
Baseline, mean (SD)	116.1 (83.6)	122.1 (84.8)	107.8 (62)	104.4 (62.5)
SDMT	(*n* = 38)	(*n* = 32)	(*n* = 31)	(*n* = 31)
mean (SD)	47.6 (13.1)	40.4 (14.3)	39.1 (12.7)	44.2 (21.5)
cMAS (0–48) **	(*n* = 19)	(*n* = 18)	(*n* = 21)	(*n* = 11)
median, (IQR)	5.0 (1.0–12.0)	7.5 (3.0–14.0)	11 (5.0–18.0)	12 (3.0–13.0)
PROMIS Sleep	(*n* = 37)	(*n* = 31)	(*n* = 29)	(*n* = 30)
mean (SD)	54.6 (6.0)	56.3 (6.4)	55.8 (6.1)	53.9 (7.2)
PROMIS Anxiety	(*n* = 38)	(*n* = 31)	(*n* = 29)	(*n* = 30)
mean (SD)	50.3 (8.9)	48.1 (7.3)	52.0 (8.4)	50.9 (8.8)
PROMIS Depression	(*n* = 37)	(*n* = 31)	(*n* = 26)	(*n* = 30)
mean (SD)	49.6 (9.2)	48.9 (9.2)	53.7 (8.6)	50.2 (9.6)

THC: delta-9-tetrahydrocannabinol, CBD: cannabidiol, THC&CBD: delta-9-tetrahydrocannabinol and cannabidiol, NRS: numerical rating scale 0–10, *n*: eligible in each group, NPSI: Neuropathic Pain Symptom Inventory (Total score 0–10), *n* = eligible replies, ^§^ Expectation of pain/spasticity relief (0= no relief, 10 =total relief) IQR: interquartile range (25–75%), Quality of life 5Q-5L-5D: questionnaire. index from −0.624–+1, PASAT: the Paced Auditory Serial Addition Test (PASAT, 3-s rate), TMT A/TMT B: Trail-making test A and B, SDMT: Symbol Digit Modality test, cMAS: combined Modified Ashworth Scale, PROMIS: (questionnaire short form sleep (T-score 31.7–76.1), anxiety (T-score 39.1–82.7), depression (T-score 38.4–80.3)), * Patients with pain >3, ≤9 at baseline, ** Patients with spasticity >3 at baseline.

**Table 3:** Pain outcomes (patients with baseline pain >3, ≤9) and spasticity outcomes (patients with baseline spasticity >3).

Primary Outcome (Pain)	Placebo *n* = 35	THC *n* = 24	CBD *n* = 27	THC&CBD *n* = 28	*p* Interaction between THC and CBD	*p* THC	*p* CBD
LOCF Change in pain (0–10 NRS) mean (SD)	−1.8 * (1.8)	−1.4 * (2.0)	−1.4 * (1.6)	−1.6 * (1.8)			
Effect size for the isolated cannabinoids (95%CI) ^^^		THC 0.83 (−0.61–0.75)	CBD 0.71 (−0.55–0.81)		0.31	0.83	0.71
Effect size of each treatment group ^#^ (95%CI)		0.42 (−0.54–1.38)	0.45 (−0.47–1.38)	0.16 (−0.75–1.08)	One-way *p* = 0.74		
BOCF Change in pain (0–10 NRS) mean (SD)	−1.9 * (1.8)	−1.3 * (2.0)	−1.4 * (1.6)	−1.5 * (1.9)	0.28	0.57	0.62
Difference NPSI (no = 101) mean (SD) The total difference Burning pain Pressing pain Paroxysmal pain Evoked pain Paresthesia/dysesthesia	−0.8 * (1.6) −1.7 * (4.0) −0.8 (2.5) −1.3 * (2.1) −0.7 (2.2) −0.4 (3.4)	−1.3 * (1.7) −1.1 (3.5) −1.2 (2.3) −1.5 * (3.0) −0.8 (2.8) −1.5 * (2.7)	−1.5 * (13.4) −1.3 (3.2) −1.1 * (2.3) −2.0 * (2.8) −1.4 * (2.5) −1.4 * (2.7)	−0.8 * (1.3) −1.5 (4.0) −2.1 * (2.1) −1.3 * (2.8) −0.5 (1.7) −0.4 (2.7)	0.07 0.53 0.54 0.37 0.28 0.06	0.74 0.80 0.16 0.61 0.33 0.96	0.58 0.96 0.26 0.53 0.51 0.89
50% pain reduction, *n* (%)	8 (22.9)	5 (20.8)	4 (14.8)	7 (25.0)	Fisher 0.81		
30% pain reduction, *n* (%)	16 (45.7)	7 (29.2)	11 (40.7)	14 (50.0)	Fisher 0.47		
Pain relief (1–10) ^$^ Median (IQR)	(*n* = 27) 2.0 (1.0–5.0)	(*n* = 15) 5.0 (1.0–7.0)	(*n* = 25) 1.0 (1.0–3.0)	(*n* = 20) 3.0 (1.0–6.5)	KW 0.19		
Primary outcome (spasticity)	Placebo *n* = 29	THC *n* = 25	CBD *n* = 26	THC&CBD *n* = 20	*p* Interaction between THC and CBD	*p* THC	*p* CBD
LOCF Change in spasticity (0–10 NRS) mean (SD)	−1.7 * (2.3)	−1.5 * (2.0)	−1.3 * (1.9)	−1.6 * (2.7)			
Effect size for the isolated cannabinoids (95%CI) ^^^		THC 0.95 (−0.92–0.86)	CBD 0.61 (−0.70–1.01)		0.51	0.95	0.67
Effect size of each treatment group ^#^ (95%CI)		0.24 (−0.67–1.45)	0.46 (−0.74–1.65)	0.10 (−1.18–1.39)	One-way *p* = 0.89		
BOCF Change in spasticity (0–10 NRS) mean (SD)	1.8 * (2.6)	1.5 * (1.9)	1.3 * (1.9)	1.5 * (2.8)	0.65	0.88	0.49
cMAS (0–48) Difference, median (IQR)	(*n* = 15) 0.0 (−5.0–0.0)	(*n* = 13) −1.0 (−4.0–0.0)	(*n* = 15) −4.0 * (−8.0–0.0)	(*n* = 10) −2.0 (−8.0–0.0)	KW 0.53		
50% spasticity reduction, *n* (%)	7 (24.1)	7 (28)	4 (15.4)	6 (30)	Fisher 0.63		
30% spasticity reduction, *n* (%)	10 (34.5)	12 (48.0)	11 (42.3)	11 (55.0)	Fisher 0.53		
Spasticity relief ^$^ (1–10) Median (IQR)	(*n* = 21) 2.0 (1.0–4.0)	(*n* = 12) 3.0 (1.0–6.0)	(*n* = 20) 1.0 (1.0–4.0)	(*n* = 12) 2.0 (1.0–4.0)	KW 0.93		

THC: delta-9-tetrahydrocannabinol, CBD: cannabidiol, THC&CBD: delta-9-tetrahydrocannabinol and cannabidiol, NRS: numerical rating scale 0–10, *n*: eligible in each group, LOCF: last observation carried forward. A negative mean value equals a lower mean score at the endpoint, BOCF: baseline observation carried forward. NPSI: Neuropathic Pain Symptom Inventory (Total score 0–10), IQR: interquartile range (25–75%), KW: Kruskal–Wallis *p*-value, cMAS: combined Modified Ashworth Scale (0–48, a negative value equals less spasticity at endpoint) * *p*-value < 0.05 (paired *t*-test within groups, baseline vs. endpoint), ^^^ Effects size on isolated cannabinoids using results from THC or CBD containing groups (two-way ANOVA) ^#^ Effects size based on one-way ANOVA with placebo as constant, ^$^ Pain/spasticity relief last week of treatment.

**Table 4:** Secondary and tertiary outcomes and measurements assessed for all patients (both pain and spasticity).

	Placebo	THC	CBD	THC&CBD	*p*	*p*	*p*
**Total**	40	32	31	31	Interaction THC and CBD	THC	CBD
Quality of life 5Q-5L-5D Index difference, mean (SD)	0.06 (0.1)	0.03 (0.1)	0.07 (0.2)	0.08 (0.2)	0.47	0.66	0.44
Impact on sleep (NRS0-10) Mean difference (SD)	−1.8 * (2.3)	−1.7 * (2.2)	−1.3 * (1.9)	−1.6 * (1.9)	0.60	0.81	0.33
Dropouts before week 5 *n* (%) ^#^	3 (7.5)	7 (21.9)	1 (3.2)	5 (16.1)	Fisher: 0.09		
Daily dose at endpoint (LOCF) Daily dose, median (IQR) Reaching full dose *n* (%)	9.0 (9.0–9.0) 32 (80.0)	9.0 (5.9–9.0) 16 (50.0)	9.0 (9.0–9.0) 24 (77.4)	7.0 (4.9–9.0) 12 (38.7)	KW <0.01		
PROMIS Sleep, *n* Difference, mean (SD)	(*n* = 28) 5.0 * (6.6)	(*n* = 19) 5.5 * (7.4)	(*n* = 27) 2.5 * (5.7)	(*n* = 22) 5.4 * (7.2)	0.39	0.21	0.26
PROMIS Anxiety, *n* Difference, mean (SD)	(*n* = 28) 4.3 * (7.3)	(*n* = 20) 1.5 (9.2)	(*n* = 27) 4.4 * (8.9)	(*n* = 22) 5.0 * (7.8)	0.32	0.57	0.35
PROMIS Depression, *n* Difference, mean (SD)	(*n* = 28) 1.8 (6.8)	(*n* = 20) −0.3 (11.0)	(*n* = 24) 2.7 (6.5)	(*n* = 22) 3.2 (7.6)	0.44	0.61	0.22
9-Hole PEG test difference *n* dom/non-dom diff. dom hand median (IQL) Diff. non-dom hand	(*n* = 30/28) 1.3 (−0.7–3.0) 0.4 (−1.2–4.0)	(*n* = 22/21) 1.4 (−0.9–4.0) 1.2 (−1.6–3.6)	(*n* = 27/27) −0.1 (−1.1–2.4) 0.0 (−4.2–1.5)	(*n* = 25/23) 0.6 (−1.0–2.0) −0.4 (−2.5–1.5)	KW 0.70 0.21		
PASAT (No. correct 0–60) *n* responders Difference, mean (SD)	(*n* = 27) −3.7 * (6.5)	(*n* = 20) −0.6 (9.6)	(*n* = 24) −1.8 (9.8)	(*n* = 20) 1.6 (6.5)	0.96	0.06	0.25
TMT A (s) Difference, mean (SD)	(*n* = 29) 5.0 (24.4)	(*n* = 23) 4.2 (10.6)	(*n* = 28) 0.6 (23.7)	(*n* = 26) 7.4 (21.5)	0.88	0.09	0.77
TMT B (s) Difference, mean (SD)	(*n* = 30) −5.8 (57.6)	(*n* = 22) −14.1 (49.0)	(*n* = 28) −17.9 * (43.5)	(*n* = 24) 13.2 (48.0)	0.08	0.09	0.35
SDMT mean (SD)	(*n* = 33) −1.1 (5.9)	(*n* = 25) 1.1 (7.2)	(*n* = 29) −1.9 (7.3)	(*n* = 27) −3.1 (11.1)	0.26	0.73	0.12
Blinding assessment *n* Active treatment, *n* (%) Placebo *n* (%) Don’t know, *n* (%)	(*n* = 36) 16 (44.4) 16 (44.4) 4 (11.1)	(*n* = 26) 14 (53.8) 9 (34.6) 3 (11.5)	(*n* = 30) 5 (16.7) 19 (63.3) 6 (20.0)	(*n* = 28) 18 (64.3) 7 (25.0) 3 (10.7)	Fisher 0.012		
Reasons ‘Guess of treatment’(>than one option was allowed) Efficacy on pain, *n* (%) Efficacy on spasticity, *n* (%) Adverse events, *n* (%) Others/don’t know, *n* (%)	19 (52.7) 19 (52.7) 12 (33.3) 17 (47.2)	13 (50.0) 11 (42.3) 10 (38.5) 6 (23.1)	19 (63.3) 15 (50.0) 8 (26.7) 13 (43.3)	11 (39.3) 5 (17.9) 14 (50.0) 10 (35.7)			

THC: delta-9-tetrahydrocannabinol, CBD: cannabidiol, THC&CBD: delta-9-tetrahydrocannabinol and cannabidiol, NRS: numerical rating scale 0–10, *n* = eligible replies, Daily dose at endpoint: 1–9 capsules/day, PROMIS: questionnaire short form sleep (T-score 31.7–76.1), anxiety (T-score 39.1–82.7), depression (T-score 38.4–80.3)), Quality of life 5Q-5L-5D, questionnaire (index from −0.624–+1), PASAT: The Paced Auditory Serial Addition Test (PASAT, 3 s rate). TMTA/TMTB: Trail-Making Test A and B, SDMT: Symbol Digit Modality Test, cMAS: combined Modified Ashworth Scale, Fisher: Fisher’s *p*-value, KW: Kruskal–Wallis *p*-value, (For “impact on sleep” a negative mean value equals a lower mean score at the endpoint. For PROMIS, 9-hole PEG, PASAT, TMTA, TMTB, and SDMT the difference is calculated as baseline minus endpoint, and a positive value means a lower (“better”) score at endpoint). ^#^ Not included in PP-analysis, * *p*-value < 0.05.

**Table 5:** Adverse events reported in the trial.

	Placebo *n* = 40	THC *n* = 32	CBD *n* = 31	THC&CBD *n* = 31
1. Mouth dryness, *n* (%), *p*	11 (27.5)	17 (53.1) 0.03	9 (29.0) 0.89	21 (67.7) <0.01
2. Headache *n* (%), *p*	13 (32.5)	15 (46.9) 0.21	10 (32.3) 0.98	13 (41.9) 0.41
3. Depressive thoughts *n* (%), *p*	4 (10.0)	2 (6.25) 0.57	4 (12.9) 0.70	2 (6.5) 0.59
4. Nightmare *n* (%), *p*	1 (2.5)	1 (3.1) 0.87	0 (0.0) 0.38	2 (6.5) 0.41
5. Euphoria *n* (%), *p*	1 (2.5)	5 (15.6) 0.05	1 (3.2) 0.86	4 (12.9) 0.09
6. Dizziness *n* (%), *p*	12 (30.0)	19 (59.4) 0.01	5 (16.1) 0.17	18 (58.1) 0.02
7. Tinnitus *n* (%), *p*	4 (10.0)	6 (18.8) 0.29	4 (12.9) 0.70	4 (12.9) 0.70
8. Anxiety *n* (%), *p*	1 (2.5)	3 (9.4) 0.21	2 (6.5) 0.41	3 (9.7) 0.19
9. Nervousness *n* (%), *p*	3 (3.5)	2 (6.3) 0.84	3 (9.7) 0.74	5 (16.1) 0.25
10. Hallucinations *n* (%), *p*	1 (2.5)	3 (9.4) 0.21	3 (9.7) 0.41	0 (0) 0.38
11. Fatigue/drowsiness, *n* (%), *p*	14 (35.0)	17 (53.1) 0.12	14 (45.2) 0.39	17 (54.8) 0.10
12. Palpitations *n* (%), *p*	0 (0)	6 (18.8) 0.004	2 (6.5) 0.10	3 (9.7) 0.04
13. Flushing of the face *n* (%), *p*	3 (7.5)	6 (18.8) 0.15	1 (3.2) 0.44	2 (6.5) 0.86
14. Stomach ache *n* (%), *p*	3 (7.5)	8 (25.0) 0.04	6 (19.4) 0.14	6 (19.4) 0.14
15. Nausea *n* (%), *p*	4 (10.0)	6 (18.8) 0.29	5 (16.1) 0.44	11 (35.5) 0.01
16. Diarrhea *n* (%), *p*	2 (5.0)	3 (9.4) 0.47	0 (0) 0.21	7 (22.6) 0.03
17. Muscle pain *n* (%), *p*	10 (25.0)	6 (18.8) 0.53	4 (12.9) 0.20	6 (19.4) 0.57
18. Visual disturbance *n* (%), *p*	3 (7.5)	5 (16.6) 0.28	2 (6.5) 0.86	3 (9.7) 0.74
19. “Other” AEs *n* (%), *p*	3 (7.5)	7 (21.9) 0.08	3 (9.7) 0.74	14 (45.2) <0.01
SAR/SUSARS *n*, (%)	0 (0)	2 (6.3)	1 (3.2)	0 (0)
SAE	2 (5.0)	2 (6.5)	0 (0)	0 (0)

THC: delta-9-tetrahydrocannabinol, CBD: cannabidiol, THC&CBD: delta-9-tetrahydrocannabinol and cannabidiol, *n* = number of registration, SAR/SUSARS: serious adverse reactions/suspected unexpected serious adverse reaction. *p*-value (treatment vs. placebo), SAE: serious adverse events, all were due to infections, and none were related to the study medication.

## Data Availability

Data is contained within the article and Supplementary Material.

## Supplementary Materials

The following supporting information can be downloaded at: https://www.mdpi.com/article/10.3390/ph16081079/s1, Figure S1: Neuropathic pain (left) and spasticity (right) presented as means week-by-week in the per-protocol population, MIXED method analysis. Figure S2: Scatterplot illustrating the relative change in percentages for pain and spasticity for patients fulfilling both the inclusion criteria for pain AND spasticity. Table S1: Adverse events “other”. Figure S3: Showing adverse events in percentage for each group. Figure S4: Study visits and contents of different phases during the study.
