# Supplementary material for: Cannabis-Based Medicine for Neuropathic Pain and Spasticity—A Multicenter, Randomized, Double-Blinded, Placebo-Controlled Trial
Source: Pharmaceuticals (Basel). 2023 Jul 28;16(8):1079. doi: 10.3390/ph16081079 (PMC10459421; doi:10.3390/ph16081079)
Supplement: Supplementary file 1 [file pharmaceuticals-16-01079-s001.zip › pharmaceuticals-2525802-supplementary.pdf]

Supplementary Materials

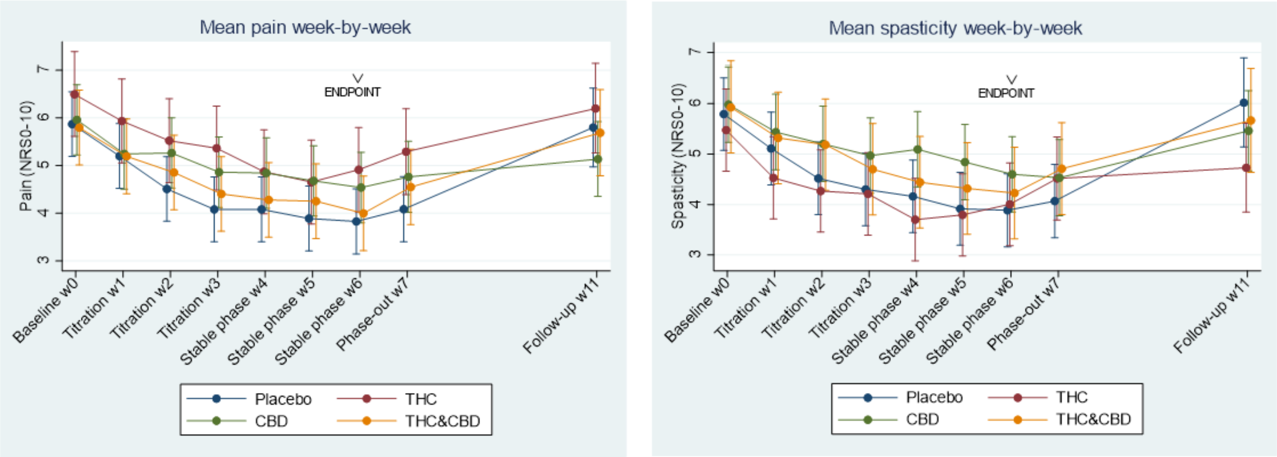

Figure S1. Neuropathic pain (left) and spasticity (right) presented as means week-by-week in the per-protocol population, MIXED method analysis.

Legend Figure S1: Weekly intensity of neuropathic pain and spasticity, stable week 6 is the endpoint measurement, week 7 is phaseout, and follow-up registration was performed 4 weeks after treatment. THC: delta-9-tetrahydrocannabinol, CBD: cannabidiol, THC&CBD: delta-9-tetrahydrocannabinol and cannabidiol, NRS: numerical rating scale 0–10.

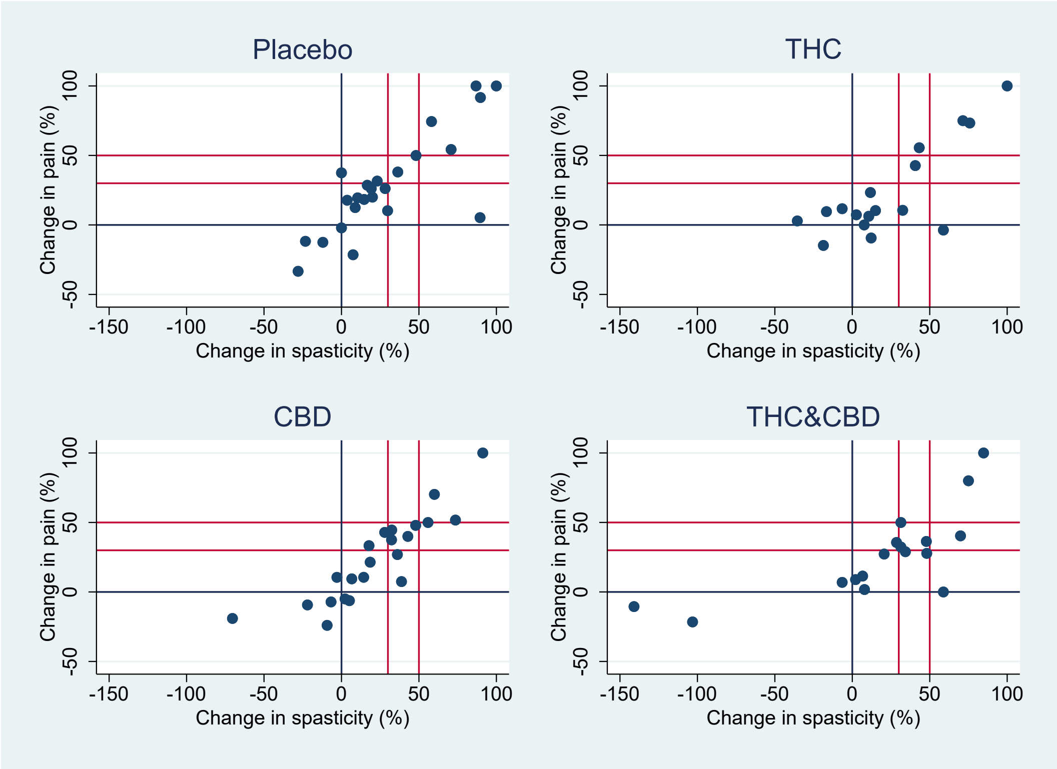

Figure S2. Scatterplot illustrates the relative change in percentages for pain and spasticity for patients fulfilling both the inclusion criteria for pain AND spasticity.

Legend Figure S2. THC: delta-9-tetrahydrocannabinol, CBD: cannabidiol, THC&CBD: delta-9-tetrahydrocannabinol and cannabidiol. A Spearman's rho (ϱ) and *p*-values showed a strong positive correlation for the difference in pain and spasticity for all patient groups that had both pain and spasticity (placebo ϱ 0.75,

$p < 0.001$, THC ϱ 0.64, $p = 0.005$, CBD ϱ0.91, $p < 0.001$, THC&CBD ϱ0.75, $p < 0.001$). Vertical red lines are 30% and 50% change in spasticity. Horizontal red lines are 30% and 50% change in pain, respectively. The blue lines (horizontal and verticals are the "0"-lines.) Each dot reflects one subject's change in pain and spasticity, respectively.

Table S1. Adverse events "other".

Treatment	Adverse Event(s)
Placebo (*n* = 3)	Sleep disturbance, joint pain<br>Prolonged bladder emptying, urge to urinate at night, stress incontinence, restless night's sleep<br>Pain on the outside of the thighs and hips. General fatigue in lower legs and arms (evening/night)
THC (*n* = 7)	Increased appetite, abdominal pain, and nausea<br>Exacerbation of spasms and affected (poorer) standing function<br>Affected "internal thermostat"<br>Stinging sensation in the hands and decreased desire for exercise<br>Affected short-term memory<br>Negative emotions are more overwhelming, thoughts "flying"<br>Instability and gait disturbance, stiffness, uncontrolled motions, incontinence
CBD (*n* = 3)	Itching<br>Pain—Gallstone attack<br>Flatulence
THC&CBD (*n* = 14)	Obstipation<br>Confusion/affected ability to concentrate<br>Affected "overview" (short term)<br>Affected (poorer) gait function and quality of life<br>Affected (poorer) gait function and decreased appetite<br>Eye pain, cramps in the calves and feet, affected (poorer) short-term memory, feeling "strange"<br>"Cheese bell" sensation<br>Increased appetite and weight gain<br>Bad mood<br>Feeling stoned, with red eyes<br>Affected (poorer) gait function<br>Feeling irritated, especially before the next dose<br>Burning sensation in extremities (like an attack), feeling drunk, sweating

Legend Table S1. THC: delta-9-tetrahydrocannabinol, CBD: cannabidiol, THC&CBD: delta-9-tetrahydrocannabinol and cannabidiol.

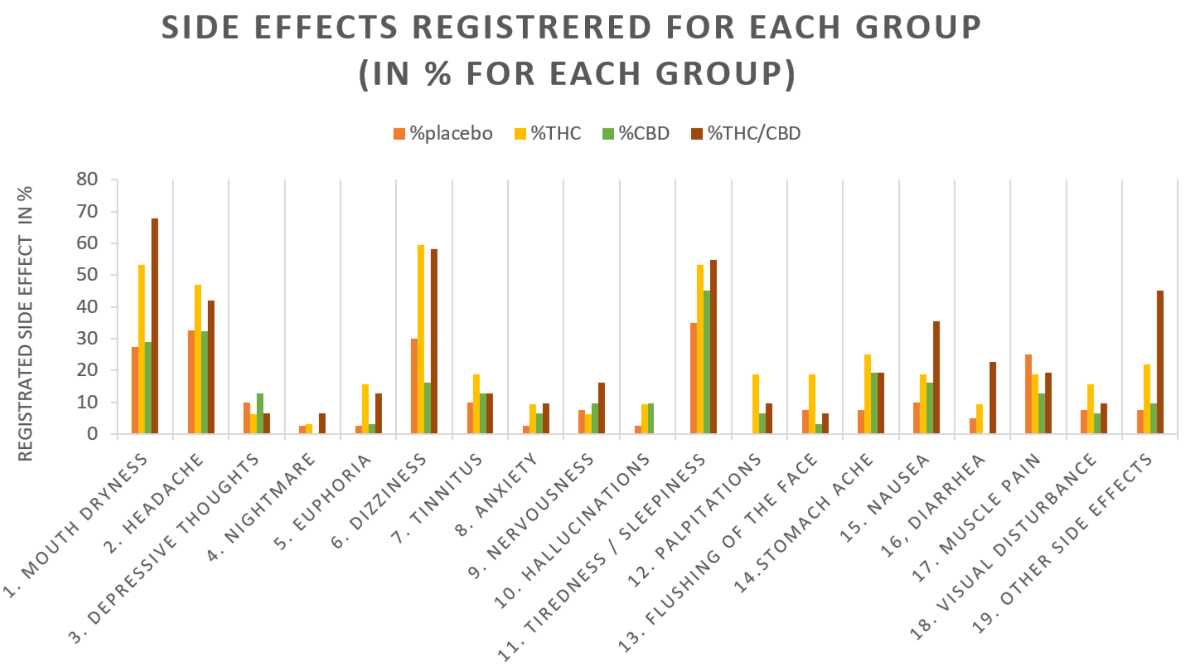

Figure S3. Showing adverse events in percentage for each group.

Legend Figure S3. Adverse events in percentage for each of the groups. A complete list of "other adverse events" is reported in table S1.

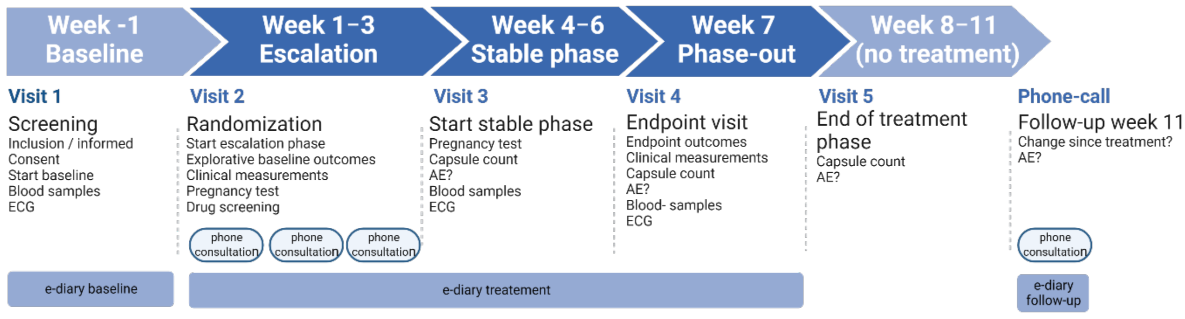

Figure S4. Study visits and contents of different phases during the study.

Legend Figure S4. Study design and content. AE: adverse event, ECG: electrocardiogram, e-diary: electronic diary. Figure created in Biorender.com.
